# Panax Notoginseng Saponins: A Review of Its Mechanisms of Antidepressant or Anxiolytic Effects and Network Analysis on Phytochemistry and Pharmacology

**DOI:** 10.3390/molecules23040940

**Published:** 2018-04-17

**Authors:** Weijie Xie, Xiangbao Meng, Yadong Zhai, Ping Zhou, Tianyuan Ye, Zhen Wang, Guibo Sun, Xiaobo Sun

**Affiliations:** 1 Beijing Key Laboratory of Innovative Drug Discovery of Traditional Chinese Medicine (Natural Medicine) and Translational Medicine, Institute of Medicinal Plant Development, Peking Union Medical College and Chinese Academy of Medical Sciences, Beijing 100193, China; ginseng123@163.com (W.X.); xbmeng@implad.ac.cn (X.M.); zydmailbox@163.com (Y.Z.); zhoup0520@163.com (P.Z.); yetianyuan2013@163.com (T.Y.); 15128475758@163.com (Z.W.); 2 Key Laboratory of Bioactive Substances and Resource Utilization of Chinese Herbal Medicine, Ministry of Education, Beijing 100193, China; 3 Key Laboratory of Efficacy Evaluation of Chinese Medicine against Glycolipid Metabolic Disorders, State Administration of Traditional Chinese Medicine, Beijing 100193, China; 4 Zhongguancun Open Laboratory of the Research and Development of Natural Medicine and Health Products, Beijing 100193, China

**Keywords:** antianxiety, antidepression, panax notoginseng saponins, network pharmacology, review

## Abstract

*Panax notoginseng (Burk) F. H. Chen*, as traditional Chinese medicine, has a long history of high clinical value, such as anti-inflammatory, anti-oxidation, inhibition of platelet aggregation, regulation of blood glucose and blood pressure, inhibition of neuronal apoptosis, and neuronal protection, and its main ingredients are Panax notoginseng saponins (PNS). Currently, *Panax notoginseng (Burk) F. H. Chen* may improve mental function, have anti-insomnia and anti-depression effects, alleviate anxiety, and decrease neural network excitation. However, the underlying effects and the mechanisms of *Panax notoginseng (Burk) F. H. Chen* and its containing chemical constituents (PNS) on these depression-related or anxiety-related diseases has not been completely established. This review summarized the antidepressant or anxiolytic effects and mechanisms of PNS and analyzed network targets of antidepressant or anxiolytic actions with network pharmacology tools to provide directions and references for further pharmacological studies and new ideas for clinical treatment of nervous system diseases and drug studies and development. The review showed PNS and its components may exert these effects through regulating neurotransmitter mechanism (5-HT, DA, NE), modulation of the gamma-amino butyric acid (GABA) neurotransmission, glutamatergic system, hypo-thalamus-pituitary-adrenal (HPA) axis, brain-derived neurotrophic factor (BDNF), and its intracellular signaling pathways in the central nervous system; and produce neuronal protection by anti-inflammatory, anti-oxidation, or inhibition of neuronal apoptosis, or platelet aggregation and its intracellular signaling pathways. Network target analysis indicated PNS and its components also may have anti-inflammatory and anti-apoptotic effects, which leads to the preservation of brain nerves, and regulate the activity and secretion of nerve cells, exerting anti-depression and anxiolytic effects, which may provide new directions for further in-depth researches of related mechanisms.

## 1. Introduction

Depression is a chronic and recurrent syndrome of mood disorder with significant and lasting low mood [1,2,3,4]. Clinical depressed patients are characteristic of its disproportionate, emotional depression, being not commensurate with their situation, low self-esteem, and even pessimism, including depressed mood from depression to grief. Some may have suicide attempts or behaviors [5,6]; in severe cases, there may be hallucinations, delusions, and other psychotic symptoms [7]. The pathogenesis and etiology of still remain unknown [8,9,10,11,12,13,14]. However, today’s knowledge proposes multiple neuronal and hormonal systems involved in the pathophysiology of the disease. Current evidence suggests that the occurrence of depression may be related to a reduced secretion of neurotransmitters [15,16,17], such as dopamine (DA), norepinephrine (NE), and serotonin (5-HT), neuronal apoptosis [14,18,19], inflammation [20,21,22,23], intestinal flora [18], and other factors [16,18,24,25]. There are currently four classes of antidepressant drugs and atypical antidepressants [25,26]. However, these drugs have undesirable side effects, with high relapse rates and a long onset of therapeutic action. Therefore, critically important is the development of efficient and safe drugs for the treatment of depression.

*Panax notoginsen (Burk) F. H. Chen* (Sanqi in Chinese) is a commonly used Chinese medicine, the root of which has been used for the treatment of hemoptysis, hemostatic, and hematoma in China and other Asian countries for its hemostatic and cardiovascular effects for more than several hundred years [26,27,28]. To the best of our knowledge, over 200 chemical constituents have been isolated from *Panax notoginseng (Burk) F. H. Chen*, but the main ingredients are Panax notoginseng saponins (PNS) [29], being classified into four types: protopanaxadiol, protopanaxatriol, ocotilloltype, and oleanolic acid constituents. There are others reported to be flavonoids, cyclopeptides, saccharides, and inorganic elements [30]. Pharmacological studies have shown that notoginseng and its extracts have many functions, such as anti-inflammatory [16,20,31,32], anti-oxidation, inhibition of platelet aggregation, regulation of blood glucose [27,33,34] and blood pressure [35,36,37], inhibition of neuronal apoptosis [14,38,39], and neuronal protection [30].

Recent studies have found that PNS have good preventive and therapeutic effects on neurological diseases of the brain [28], especially in antidepressant [26,27,28] and anti-anxiety effects [25,28,40]. The preliminary determination was that anti-depressant and anti-anxiety effects may occur by increasing the levels of the brain monoamine neurotransmitters 5-hydroxytryptamine (5-HT) [41,42] and norepinephrine (NE) in the central nervous system. Experiments also have shown that PNS modulates Na^+^ currents and Ca^2+^ concentrations, and increases nestin and brain-derived neurotrophic factor (BDNF) [14,19,29,43,44,45,46,47]. Moreover, PNS or single compounds exerted antidepressant-like effects by regulating the release of interleukin (IL)-1β, IL-6, and tumor necrosis factor (TNF)-α, or anti-inflammatory cytokines IL-4 and IL-10 [10,20,29,31,32,36,48,49,50,51,52,53,54,55,56]. Such breakthroughs have been made to determine there are antidepressant effects on estrogen in the human body. Estrogen can act on the PI3K/Akt, MAPK/ERK, mTORC1, and other signaling pathways, and then these pathways are regulated by growth factors that protect neurons and produce estrogen-like effects on synapses. However, the role of PNS or its single compounds in normal function and in disease remains to be not much elucidated. 

Therefore, this review summarized the antidepressant or anxiolytic effects and mechanisms of PNS and analyzed network targets of antidepressant or anxiolytic actions with network pharmacology tools, to provide reference for further pharmacological studies and new ideas for clinical treatment of nervous system diseases and drug studies and development. 

## 2. Mechanisms of Antidepression

### 2.1. Panax Notoginseng Saponins

Modern pharmacological studies have found Panax notoginseng saponins (PNS) have good preventive and therapeutic effects of brain neurological diseases [26,28,57]. Ginsenosides Rg1, Rb1, Re, and notoginsenoside R1, as the representative [26,28,57], showed anti-depressant and anti-anxiety activity [28,31,35,58,59,60,61]. The evaluation methods of antidepressant effects include sucrose preference test (SPF) [62], forced swimming test (FST) [63,64,65], open field test (OFT) [62,64,66], elevated plus-maze (EPM) test, and tail suspension test (TST) [66]. 

PNS (58.6%) significantly decreased immobility time in the forced swim test with little effect on locomotion in the chronic mild stress model, and significantly improved the level of animal activity and sucrose consumption [67], which may have antidepressant-like effects [45,53,64,65]. Besides this, when PNS was digested by Snail Enzyme to 50%, it produced the best antidepressant effects, and the main active ingredient includes ginsenosides Rb1, Rb2, Rc, and Rb3 [40,60,68,69,70]. These were shown in Table 1. 

At present, the antidepressant mechanisms have been partly proved to be that PNS upregulates expression of brain-derived neurotrophic factor (BDNF) by regulating multiple upstream neural signal transduction pathways, including AC/cAMP/PKA [45,71] and mitogen-activated protein kinase channels (TrKB/MAPK/PSK) [37,72]; meanwhile, PNS regulates calmodulin kinase channel (Ca^2+^/CaM/CaMK), reduces internal concentration of Ca^2+^ in nerve cells [73], and promotes the release of neurotransmitters 5-HT, NE, and DA [27,32,41,52]. Thereby, PNS exerted its antidepressant-like effect. These are shown in Table 1.

Besides this, both PNS and ginseng total saponin (GTS) have significant antidepressant effects on LPS-induced murine CD-1 mouse models [19,52,53,74] by reducing mRNA levels for IL-1β, IL-6, TNF-α, and indoleamine 2,3-dioxygenase (IDO) in hippocampus [52]. These were shown in Table 1.

### 2.2. Single Component: Rg1

Among Panax notoginseng saponins, ginsenoside Rg1, Rb1, Rc, and Rb3 are important representative components of PNS antidepressant, belonging to the original ginseng diol type saponins, and the contents of ginsenoside Rb3 and Rc were 17.66% and 9.32%, respectively [25,27,33].

By conducting open field tests (OFT), tail suspension tests (TST), forced swimming tests (FST), or sugar consumption tests, ginsenoside Rg1 showed antidepressant effect in chronic mild stress (CMS)- or chronic unpredictable mild stress (CUMS)-induced animal models of Depression [45,64,67,80]. Ginsenoside Rg1 significantly reversed increase of serum corticosterone induced by CMS, increased the phosphorylation of cAMP response element-binding protein (CREB) in rat cerebral amygdala induced by CUMS, up-regulated expression of BDNF in the hippocampus [32,43,45], and down-regulated serum corticosterone lever [43]. It also regulates the hyperthyroidism of HPA axis in depression, plays a positive role in activating BDNF and protein kinase [43,74], and also improves the neuronal status, and increases the number of neurons and dendritic spine density [32,43,45], producing antidepressant effect. 

Moreover, ginsenoside Rg1 could up-regulate the expression of connexin, improve the gap junctional connectivity of astrocytes in the prefrontal cortex, decrease the permeability, increase the ultrastructure, and reduce the depression-like behavior of rats induced by CUMS [79]. As present studies found that Ly6C (hi) blood mononuclear cells can be used as anti-depressant regulatory targets [10], Rg1 selectively suppressed Ly6C hi monocytes recruitment to the inflamed mice brain [10], Rg1 pretreatment on activated THP-1 monocytes retarded their ability to trigger CCL2 secretion from co-cultured U251 MG astrocytes, and CCL2-triggered p38/MAPK and PI3K/Akt activation were blocked by Rg1 [10,81]. Besides this, Rg1 suppressed depression-like behaviors in CUMS model rats by reducing contents of Glu and Asp in hippocampus and increasing GABA and Tau content, exerting antidepressant effects [67].

### 2.3. Single Component: Rb1

Ginsenoside Rb1 and ginsenoside K, its metabolite, could activate 5-HT2AR, and may also up-regulate the mRNA expression of 5-HT2AR, which showed anti-depressant effects on ovariectomized rat model carried with ovariectomy, whereas the anti-depression effect of Rb1 and ginsenoside K may be reversed by the 5-HT2AR antagonist (ritanserin), which generally indicates that Rb1 has a similar-neurotransmitter of 5-HT activation effect [42]. These were shown in Table 1.

### 2.4. Single Component: Rb3

Ginsenoside Rb3 had significant anti-immobility effects in mice in the forced swim and tail suspension tests, and reduced the number of escape failures in the learned helplessness procedure by using the forced swimming test, tail suspension test, and learned helplessness procedure. Biochemical variations (i.e., brain-derived neurotrophic factor and the monoamine neurotransmitters 5-hydroxytryptamine, dopamine, and norepinephrine) are mainly involved in Rb3’s antidepressant-like effects [25,27]. Using GABA_A_ receptor agonist muscimol was similar to that of ginsenoside Rb3, which indicated that the activation of the GABA_A_ receptor is correlated with the neuroprotective mechanisms of ginsenoside Rb3 [25,27,86]. These were shown in Table 1.

### 2.5. Single Component: Rg3

Ginsenoside Rg3 exerted a significant antidepressant effect on LPS-induced mouse model by immunomodulatory and anti-inflammatory effects [19,53,74], which decreased levels of IL-6, TNF-α in plasma and indoleamine-2,3-deoxygenates (IDO) expression in the hippocampus, reduced or inhibited the turnover of the tryptophan-serotonin (5-HT) in the hippocampus, regulated the secretory activity of microglia microglial cells and the transcription of NF-kappa-B in the nucleus, and repaired tryptophan-kynurenine metabolic balance, thereby weakening the depressant-like behavior or symptoms. In addition, Rg3 exerted an anti-depressant effect on chronic social defeat stress (CSDS)-induced mice model, but had no significant effect on animal activity and enhanced BNDF expression [74]. These were shown in Table 1.

Besides this, ginsenoside Rg5 induced antidepressant effect by inhibiting Trk and AchE activity and upregulating BDNF expression in chronic social defeat stress (CSDS)-induced model mice [85]. Ginsenoside Re regulated the central adrenergic system, inhibited tyrosine hydroxylase (TH) expression in the locus coeruleus, blocked the decrease of BDNF in hippocampus, and regulated the secretion of corticosterone in the HPA axis, thus alleviating and regulating the synthesis of depression or anxiety symptoms [44]. These were shown in Table 1.

## 3. Mechanisms of Antianxiety

At present, representatives of *Panax notoginseng (Burk) F. H. Chen*, stem and leaf soap components or part of ginsenosides that have been shown to have anxiolytic effects are ginsenoside Rb1, Re, PPD [40,83,87,88], ginsenoside Rh1 [40], and ginsenoside Rg3 [27,53,74,84]. In chronic mild stress (CMS)-induced rats, l-DOPA-induced running behavior in mice or 5-HTP-induced head-twitch tests, PNS decreased immobility time, increased the levels of 5-hydroxytryptamine, dopamine, and noradrenaline, reduced basal [Ca^2+^]_i_ levels in cultured neurons, which were mediated by modulation of brain monoamine neurotransmitters and intracellular Ca^2+^ concentration, producing anti-anxiety and antidepressant effect [26,28,89]. These are shown in Table 2.

Ginsenoside Rb1 reduced the anxiety index, increased the risk assessment, reduced grooming behaviors in the EPM test, and increased the total number of line crossings of an open field in single prolonged stress (SPS)-induced model and in model rats of post-traumatic stress disorder (PTSD) by increasing the expression of neuropeptide Y induced by SPS-induced decrease in hypothalamic; increase the expression of tyrosine hydroxylase in locus coeruleus and decrease the mRNA expression of BDNF in hippocampus. 

At the same time, ginsenoside Rb1 and Re’s intestinal metabolites PPD and K, ginsenoside Rh1 can increase arm opening time in EPM and reduce the serum levels of adrenone IL-6, and the role of γ-aminobutyrate-A (GABA A) receptor(s) exert anxiolytic effects [27,40]. Pseudoginsenoside-F(11) greatly ameliorated the anxiety-like behaviors induced by MA, shortened MA-induced prolonged latency, decreased the error counts, significant decreases in the contents of dopamine (DA), 3,4-dihydroxyphenacetic acid (DOPAC), homovanillic acid (HVA), and 5-hydroxyindoacetic acid (5-HIAA) in the brain of MA-treated mice; and partially, but significantly, a antagonize MA-induced decreases of DA [90]. Besides, Ginsenoside Rg3, Rh2, Rg1 and Ro significantly increased both the frequency and duration of open arm entries, indicating that ginsenoside Rb1 is one of the active anxiolytic components [84]. These were shown in Table 2.

## 4. Network Target Analysis

Based on reported researches of each component above, including ginsenoside Rg1, Rg3, Rg5, Rb1, Rb3, Re, Rh1, Rh2, Ro, pseudoginsenoside-F(11), ginsenoside K, and notoginsenoside R1, the structures and target properties of 12 active ingredients among them was obtained from the PubChem database (https://pubchem.ncbi.nlm.nih.gov/), shown as Table 3; then network targets of antidepressant, anxiolytic actions, or other pharmacological effects were analyzed with network pharmacology tools. In the present work, an integrated in silico approach was introduced to identify the target proteins for the active ingredients of *Panax notoginseng (Burk) F. H. Chen* (Table 3). Predictive models were used, including STITCH (http://stitch.embl.de/) and Swiss Target Prediction (http://www.swisstargetprediction.ch/), and databases were mined including Herbal Ingredients’ Targets database (http://lifecenter.sgst.cn/hit/) [93,94]. Finally, targets related to antidepressant or anxiolytic effects and other effects were determined, interacting with the selected 12 active ingredients of *Panax notoginseng (Burk) F. H. Chen* (Table 4). 

**Figure 1:**
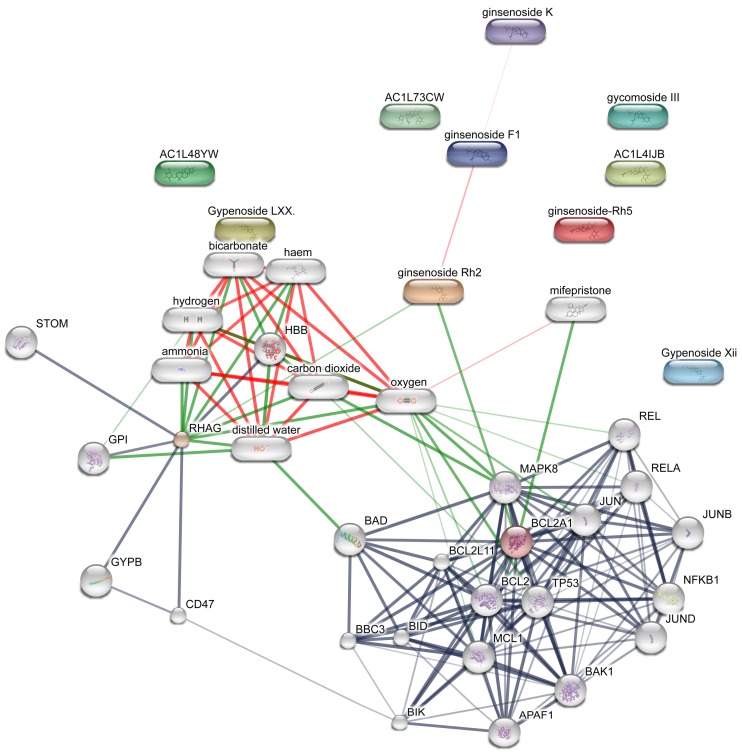
Compound-target network of *Panax notoginseng (Burk) F. H. Chen*. Edges represent protein-protein associations; protein–protein interactions are shown in grey, chemical–protein interactions in green, and interactions between chemicals in red; stronger associations are represented by thicker lines. Small nodes: protein of unknown 3D structure; large nodes: some 3D structure is known or predicted; colored nodes: query proteins and first shell of interactors; white nodes: second shell of interactors. The chemical groups and structures in the gray rectangles, such as carbon dioxide, oxygen, and HO-, suggest functional links and predictions for specific actions on with potentially acting protein targets and combined scores of predicted interactions presented by analysing the Experimental/Biochemical Data, Association in Curated Databases and Co-Mentioned in PubMed Abstracts, which illustrated the possibility of interaction between compounds and potential protein targets. Associations are meant to be specific and meaningful, i.e., Proteins jointly contribute to a shared function; this does not necessarily mean they are physically binding each other. Predicted models were constructed by STITCH, Swiss Target Prediction, and Herbal Ingredients’ Targets database.

Main predicted network targets of *Panax notoginseng (Burk) F. H. Chen* were shown in Table 4 and Figure 1. Twenty-six targets were identified for 12 active ingredients of *Panax notoginseng (Burk) F. H. Chen* with 87 interactions. The more link lines between compounds and predicted protein targets, the greater the linear density, indicating that they are more likely to interact, but specific experiments are needed to confirm. This is shown in Figure 1.

**Figure 2:**
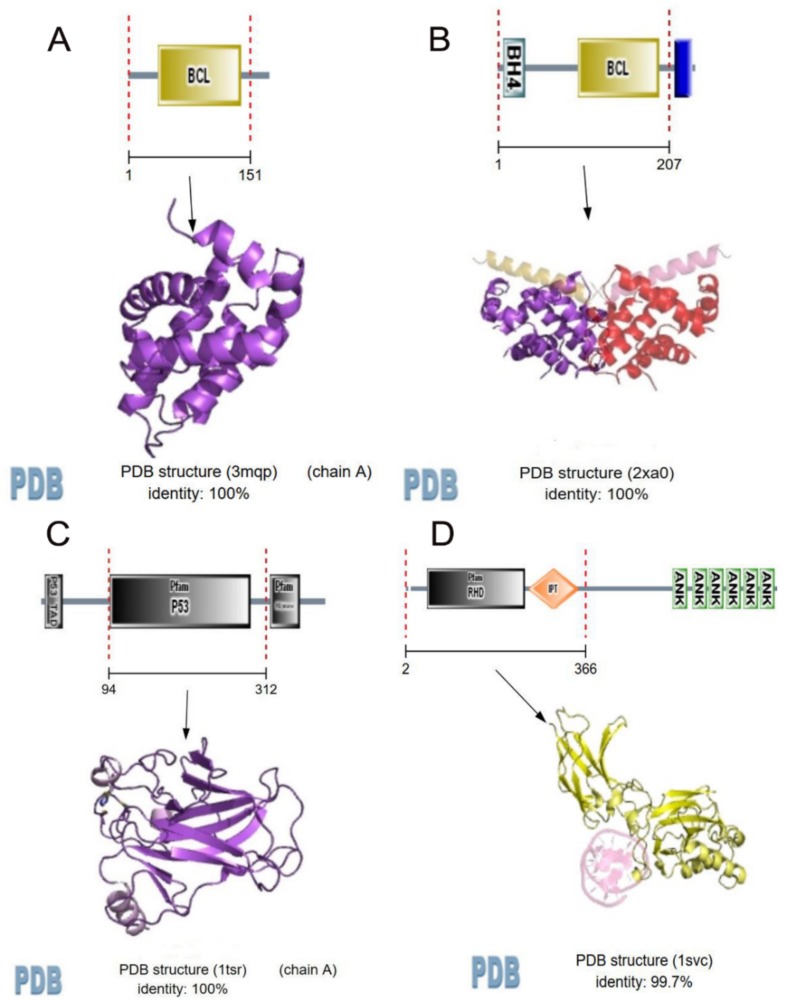
Strong association actions of compound and predicted target network of *Panax notoginseng (Burk) F. H. Chen*. (**A**) represents re-center network or functional domain of compounds associated with BCL2-A1; (**B**) represents BCL2; (**C**) represents TP53; (**D**) represents NFKB1; BCL2-A1, BCL2, NF-kappa-B, and TP53 were predicted to have strong association actions of compound and predicted target network of *Panax notoginseng (Burk) F. H. Chen*, indicating that PNS may exert anti-depression and anti-anxiety effects through inflammatory regulation and anti-apoptotic pathway. Association action models were constructed by STITCH and PDB database.

Based on the scores (Table 4), multiple therapeutic targets concerning central nervous system were mediated by the active ingredients of *Panax notoginseng (Burk) F. H. Chen*, such as BAK1, BCL2, BCL2A1, JUN, MAPK8, AKT1, NFKB1, TP53, and APAF1. Most of these targets are mainly involved in apoptosis and inflammation of vascular and central neural systems. Figure 1 and Figure 2 show that BCL2-A1, BCL2, NF-kappa-B, and TP53 was predicted to have strong association actions of compound and predicted target network of *Panax notoginseng (Burk) F. H. Chen*, indicating that Notoginseng saponins may exert anti-depression and anti-anxiety effects through inflammatory regulation and anti-apoptotic pathway. 

BCL2 suppresses apoptosis in a variety of cell systems, including neural cells and factor-dependent lymphohematopoietic, and regulates cell death by controlling the mitochondrial membrane permeability by binding to the apoptosis-activating factor (APAF-1). NF-kappa-B is a pleiotropic transcription factor present in almost all cell types, and is the endpoint of a series of signal transduction events that are initiated by a vast array of stimuli related to many biological processes, such as inflammation, immunity, differentiation, cell growth, tumorigenesis, and apoptosis. TP53 acts as a tumor suppressor in many tumor types, and induces growth arrest or apoptosis depending on the physiological circumstances and cell type, involved in cell cycle regulation as a trans-activator that acts to negatively regulate cell division by controlling a set of genes required for this process. These indicate that PNS may have anti-inflammatory and anti-apoptotic effects, which leads to the preservation of brain nerves, and regulate the activity and secretion of nerve cells, exerting anti-depression and anxiolytic effects, which may provide new directions for further in-depth researches of related mechanisms.

## 5. Conclusions

Depression is becoming the world’s fourth-largest disease, and may become the second-largest disease in 2020, second only to cardiac disease. Antidepressants can be divided into four categories: tricyclic, tetracyclic, monoamine oxidase inhibitors, and other new antidepressants, and sometimes can cause addiction or undesirable side effects. Therefore, new drugs or treatment methods are required. Future studies should consider investigating alternatives to conventional drugs or Chinese medicinal plants. These have been used for more than a thousand years with generally carry a low risk of negative side effects. *Panax notoginseng (Burk) F. H. Chen*, as traditional Chinese medicine has a long history of high clinical value, such as anti-inflammatory [16,20,31,32], anti-oxidation, inhibition of platelet aggregation, regulation of blood glucose [27,33,34] and blood pressure [35,36,37], inhibition of neuronal apoptosis [14,38,39], and neuronal protection. Currently, *Panax notoginseng (Burk) F. H. Chen* may improve mental function, have anti-insomnia and anti-depression effects, alleviate anxiety, and decrease neural network excitation [25,26].

As outlined above, we have confirmed that PNS, ginsenoside Rg1, ginsenoside Rb1, ginsenosidenRg3, ginsenoside Rh2, ginsenoside Rg5, K, and ginsenoside Re have antidepressant or anxiolytic effects, and ginsenoside Rh1and pseudoginsenoside-F(11) have anxiolytic effects. These components may exert these effects through regulating neurotransmitter mechanism (5-HT, DA, NE), modulation of the gamma-amino butyric acid (GABA) neurotransmission, glutamatergic system, hypo-thalamus-pituitary-adrenal (HPA) axis, brain-derived neurotrophic factor (BDNF), and its intracellular signaling pathways in the central nervous system; and produce neuronal protection by anti-inflammatory, anti-oxidation, inhibition of neuronal apoptosis, or platelet aggregation and its intracellular signaling pathways. 

Although, among them, some compounds have been only proved to have antidepressant or anxiolytic effects in GTS [95], the main chemical composition of *Panax notoginseng (Burk) F. H. Chen* is similar to that of ginseng; at present, less researches have been shown on the role of Panax *notoginseng (Burk) F. H*. Chen and chemical composition in anti-anxiety and anti-depression. Therefore, as a potential new drug, a systematic and in-depth study of the effects and its molecular mechanisms of anti-anxiety and anti-depression of *Panax notoginseng (Burk) F. H. Chen* is needed. 

Further work on elucidation of the structure–function relationship among saponins, understanding of multi-target network pharmacology of *Panax notoginseng (Burk) F. H. Chen* with Network Pharmacology tools, as well as developing its new clinical usage and comprehensive utilize will enhance the therapeutic potentials of *Panax notoginseng (Burk) F. H. Chen*. A clearer understanding of the mechanisms underlying the effects of *Panax notoginseng (Burk) F. H. Chen* on human cytokine/metabolic systems and on stress-induced hormonal changes could facilitate the development of a wide range of treatments for patients with psychological and physical diseases.

## Figures and Tables

**Table 1:** Phytochemicals and mechanisms with antidepressant effects in Panax notoginseng saponins (PNS).

Name	Methods	Models	Neurobehavioral Effects and Mechanisms
PNS (58.6%)	OFT; TST; FST; SPT [26,53]	CMS-induced mice; CUMS-induced rats [26,53]	↓ Immobility time in FST and TST; ↑ sucrose intake in sucrose preference test; ↑ level of animal activity; ↑ Expression of BDNF [40,60,68,69,70].
Purified PNS	SPT; OFT [45,71]	CUMS-induced SD rats [45,71]	Regulate upstream neural signal transduction pathways: AC/cAMP/PKA, TrKB/MAPK/PSK and Ca^2+^/CaM/CaMK signaling pathway; ↓ Concentration of Ca^2+^ in nerve cells; ↑ release of neurotransmitter 5-HT, DA, and NE [45,71].
PNS and GTS	FST; SPT [53,75,76,77,78].	LPS-induced KM mice; CD-1 mice [53,75,76,77,78]	↓ mRNA of IL-1β, IL-6, TNF-α, and IDO; ↓ Inflammation in the brain [53,75,76,77,78].
Ginsenoside Rg1	OFT; FST; SPT [19,45]	CMS and CUMS-induced depression Animal Model [19,45]	↓ CMS-induced increasement of corticosterone levels in serum; ↑ CUMS-induced CREB phosphorylation in the amygdala of the brain [45]; ↑ expression of BDNF [33,43]; regulate hyperactivity of the HPA axis in depressed states; ↑neuronal status, neurons quantity and density of dendritic spines [19,45].
Ginsenoside Rg1	FST; SPT [79]	CMS and CUMS-induced depression animal model [80]	↑ Expression of connexin and gap junction of astrocytes in the prefrontal cortex of the brain; ↑ dense ultrastructure; ↓ permeability and depression-like behaviors induced by CUS in rats [79].
Ginsenoside Rg1	FST; SPT [10]	Cerebral inflammatory model mice [10]	↓ Recruitment of Ly6C (hi) monocytes in inflammatory brain model mice [10,16]; activate THP-1 monocytes; blocks the feedback-regulated release of CCL2 from astroglial cells; ↓ CCL2 signaling pathways: p38/MAPK and PI3K/Akt activities [10,81].
Ginsenoside Rg1	TST; FST; SPT [15,67]	CUMS-induced depression rats [67]	↓ Contents of Glu and Asp in hippocampus, ↑ contents of GABA and Tau; ↓ depression-like Behavior in CUMS model rats [15,67]
Ginsenoside Rb1 and ginsenoside K	TST; FST [42]	CUMS-induced depression rats [42]	↑ Expression of 5-HT2AR mRNA and activate of 5-HT2AR [42]; the antidepressant effects of Rb1 and the metabolite ginsenoside K may be antagonized by 5-HT2AR antagonists (Ritanerin), indicating that Rb1 has a similar 5-HT transmitter activation effect [68,82,83].
Ginsenoside Rg3 and ginsenoside Rh2	OFT; SPT [19,53,74]	LPS-induced mice [19,53,74] and CSDS-induced mice [74]	↓ IL-6 and TNF-α in plasma and the expression of indoleamine 2,3-dioxygenase (IDO) in brain; ↓ turnover of tryptophan and 5-HT in hippocampal tissue; regulate secretory activities of microglia and transcription of NF-kappa-B in nuclear; ↑ expression of BNDF; ↓ depressive behavior or symptoms [53,74,84].
Ginsenoside Rg5	OFT; FST; SPT [85]	CSDS-induced mice [85]	↓ Trk and AchE; ↑expression of BNDF [69,85].
Ginsenoside Re	OFT; TST [44]	CMS-induced rat model [44]	Regulate the central adrenergic system; ↓ expression of TH in the locus coeruleus area; ↓ decrease of BDNF in the hippocampus, and regulates the secretion of corticosterone from the HPA axis [44].

GST, ginseng total saponin ; SPT, sucrose preference test; FST, forced swimming test; OFT, open field test; TST, tail suspension test; CMS, chronic mild stress; CUMS, chronic unpredictable mild stress; CREB, cAMP response element-binding protein; CSDS, chronic social defeat stress.

**Table 2:** Phytochemicals and mechanisms with anxiolytic effects in Panax notoginseng saponins (PNS).

Name	Methods	Models	Neurobehavioral Effects and Mechanisms
PNS	OFT; SPT [26,28]	l-DOPA-induced mice [26,28]; 5-HTP-induced rats [89]	↓ Basal [Ca^2+^]_i_ levels; ↓ immobility time; ↑ levels of 5-HT, DA and NE [26,28,89].
Ginsenoside Rb1	EPM test [44,91]	SPS model and rat model of post-traumatic stress disorder [44,91]	↓ Anxiety index;↑ Risk assessment; ↓ grooming behaviors in EPMT; ↑ total number of line crossings of an open field after SPS; ↓ SPS-induced decreasement in hypothalamic neuropeptide Y expression; ↑ in locus cerulean tyrosine hydroxylase expression; ↓ expression of BDNF [44,91].
Ginsenoside Rb1, Re; ginsenoside Rh1, PPD	EPM test [27,40]	Immobilization stress-induced ICR mice [27,40]	↑ Time spent in open arms and open arm entries in EPM tests; ↓ Immobilization stress-induced serum levels of corticosterone and IL-6; ↓ anxiolytic effect via γ-aminobutyrate A (GABA A) receptor(s) [27,40].
Ginsenoside Rg3	Two electrode voltage-clamp technique [86,92]	Xenopus laevis frogs [86,92]	↓ Anxiolytic effect via γ-aminobutyrate A (GABA A) receptor(s) [86,92].
Ginsenoside Re	FST; EPM; AAT [44]	Repeated immobilization stress-induced rats [44]	↓ Stress-induced behavioral deficits in these behavioral tests; ↓ TH expression in LC; ↓ mRNA expression of BDNF in the hippocampus; modulate central noradrenergic system in rats [44].
Ginsenoside Rg3 and ginsenoside Rh2	EPM test [27,84]	Male ICR mice [27,84]	↑ Time spent on the open arms and the number of open-arm entries; antagonize GABA/benzodiazepines [27,84].
Ginsenoside Rb1, Rg1, and Ro	EPM test [68,84]	Male ICR albino mice [68,84]	↑ Both the frequency and duration of open arm entries [68,84].
Pseudoginsenoside-F11	Light-dark box test; FST [90]	MA-induced behavioral and neurochemical toxicities in mice [90]	↓ Anxiety-like behavior induced by methamphetamine (MA); ↓ MA-induced prolonged latency; ↓ the error counts; ↓ contents of DOPAC, HVA, and 5-HIAA in the brain of MA-treated mice; antagonize decreases of DA [90].

EPM, elevated plus-maze.

**Table 3:** Active ingredients of Panax notoginseng saponins.

No.	Name	Structure
PNS-1	Ginsenoside Rg1	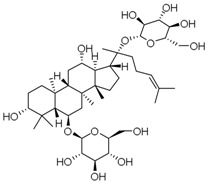
PNS-2	Ginsenoside Rg3	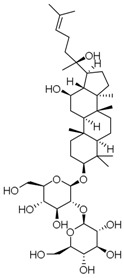
PNS-3	Ginsenoside Rg5	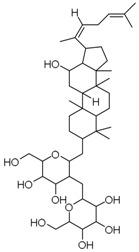
PNS-4	Ginsenoside Rb1	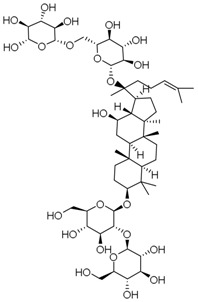
PNS-5	Ginsenoside Rb3	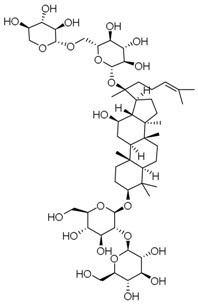
PNS-6	Ginsenoside Re	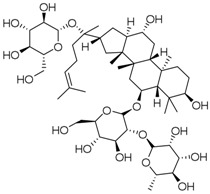
PNS-7	Ginsenoside Rh1	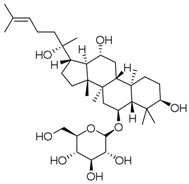
PNS-8	Ginsenoside Rh2	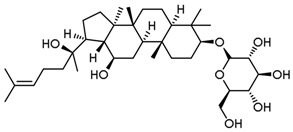
PNS-9	Pseudoginsenoside-F11	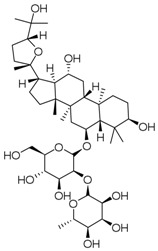
PNS-10	Ginsenoside Ro	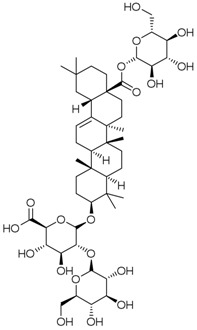
PNS-11	Ginsenoside K	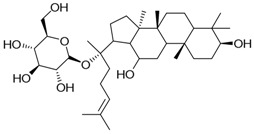
PNS-12	Notoginsenoside R1	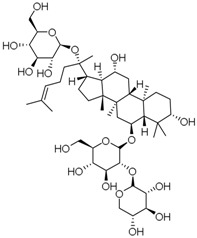

**Table 4:** Main predicted target information of *Panax notoginseng (Burk) F. H. Chen.*

Node1	Node2	Node1 Accession	Node2 Accession	Score
APAF1	BAK1	ENSP00000448165	ENSP00000353878	0.997
APAF1	BCL2	ENSP00000448165	ENSP00000329623	0.999
APAF1	BCL2A1	ENSP00000448165	ENSP00000267953	0.912
APAF1	BIK	ENSP00000448165	ENSP00000216115	0.932
APAF1	JUN	ENSP00000448165	ENSP00000360266	0.582
APAF1	MAPK8	ENSP00000448165	ENSP00000353483	0.611
APAF1	MCL1	ENSP00000448165	ENSP00000358022	0.95
APAF1	NFKB1	ENSP00000448165	ENSP00000226574	0.608
APAF1	TP53	ENSP00000448165	ENSP00000269305	0.996
BAK1	APAF1	ENSP00000353878	ENSP00000448165	0.997
BAK1	BBC3	ENSP00000353878	ENSP00000404503	0.414
BAK1	BCL2	ENSP00000353878	ENSP00000329623	0.999
BAK1	BCL2A1	ENSP00000353878	ENSP00000267953	0.987
BAK1	BIK	ENSP00000353878	ENSP00000216115	0.583
BAK1	JUN	ENSP00000353878	ENSP00000360266	0.852
BAK1	JUNB	ENSP00000353878	ENSP00000303315	0.658
BAK1	JUND	ENSP00000353878	ENSP00000252818	0.671
BAK1	MAPK8	ENSP00000353878	ENSP00000353483	0.83
BAK1	MCL1	ENSP00000353878	ENSP00000358022	0.99
